# Acknowledgment of Reviewers

**DOI:** 10.2188/jea.JE20110118

**Published:** 2011-11-05

The following scientsts assisted the journal by reviewing manuscripts during the period October 1, 2010 to September 30, 2011. We gratefully acknowledge their critical evaluation and assistance in selecting articles for publication.

**Table:** 

Adachi, Hisashi	Kurume University
Aida, Jun	Tohoku University
Anme, Tokie	Tsukuba University
Arao, Takashi	Waseda University
Babazono, Akira	Kyushu University
Cai, Guoxi	Research Institute for Humanity and Nature
Chihara, Izumi	Jichi Medical University
Doi, Yasufumi	Kyushu University
Doi, Yuriko	National Institute of Public Health
Fujita, Yasuyuki	Shimane University
Fujiwara, Nakako	Aichi Prefectural University
Fukuda, Yoshiharu	Yamaguchi Unversity
Goto, Aya	Fukushima Medical University
Hamajima, Nobuyuki	Nagoya University
Hara, Megumi	Saga University
Hashimoto, Shuji	Fujita Health University
Hata, Akira	Chiba University
Hayakawa, Kazuo	Osaka University
Hayasaka, Shinya	Hamamatsu University School of Medicine
Hayashi, Kunihiko	Gunma University
Hayashino, Yasuaki	Kyoto University
Hirose, Kaoru	Aichi Prefectural Institute of Public Health
Honda, Sumihisa	Nagasaki University
Honda, Yasushi	University of Tsukuba
Honjo, Satoshi	Fukuoka East Medical Centre
Hoshi, Keika	Kitasato University
Hoshiyama, Yoshiharu	University of Human Arts and Sciences
Hoshuyama, Tsutomu	Ushibuka-Shimin Hospital
Hosono, Satoyo	Aichi Cancer Center Research Institute
Hozawa, Atsushi	Tohoku University
Imai, Tomoko	Tokaigakuen University
Imamura, Fumiaki	Harvard School of Public Health
Imanaka, Yuichi	Kyoto University
Inoue, Shigeru	Tokyo Medical University
Ishihara, Junko	Tokyo University of Agriculture
Ishikawa, Hirofumi	Okayama University
Ishizaki, Tatsuro	Kyoto University School of Public Health
Ito, Yuri	Osaka Medical Center for Cancer and Cardiovascular Disease
Iwasa, Hajime	Tokyo Metropolitan Institute of Gerontology
Kai, Ichiro	University of Tokyo
Kakehashi, Masayuki	Hiroshima University
Kaneita, Yoshitaka	Nihon University School Medicine
Kaneko, Satoshi	Nagasaki University
Kasagi, Fumiyoshi	Radiation Effects Association
Katanoda, Kota	National Cancer Center
Kato, Noriko	National Institute of Public Health
Kawaguchi, Yoko	Tokyo Medical and Dental University
Kawamura, Takashi	Kyoto University
Kayaba, Kazunori	Saitama Prefectural University
Kikuchi, Shogo	Aichi Medciacl University
Kishimoto, Takuji	Tottori University
Kiyohara, Yutaka	Kyushu University
Kodama, Tomoko	National Institute of Public Health
Kojima, Masayo	Nagoya City University
Kokubo, Yoshihiro	National Cardiovascular Center
Kondo, Naoki	University of Yamanashi
Kondo, Takaaki	Nagoya University
Kubo, Tatsuhiko	University of Occupational and Environmental Health
Kuriki, Kiyonori	Laboratory of Public Health, School of Food and Nutritional Sciences
Kuriyama, Shinichi	Tohoku University
Kurokawa, Naoyuki	Tohoku University
Ma, Enbo	University of Tsukuba
Masaki, Motofumi	University of Nagasaki
Matsuo, Keitaro	Aichi Cancer Center Research Institute
Metoki, Hirohito	Tohoku University
Minami, Yuko	Tohoku University
Miura, Yoshihiko	Saitama Prefectural University
Miyaki, Koichi	National Center for Global Health and Medicine
Mizoue, Tetsuya	International Medical Center of Japan
Mochizuki, Yoshikatsu	Asahikawa Medical University
Mori, Mitsuru	Sapporo Medical University
Morikawa, Yuko	Kanazawa Medical University
Morita, Akemi	National Institute of Health and Nutrition
Morita, Manabu	Okayama University
Murakami, Yoshitaka	Shiga University of Medical Science
Murata, Katsuyuki	Akita University
Nagai, Masaki	Saitama Medical University
Nagano, Jun	Kyushu University
Nagata, Chisato	Gifu University
Naito, Mariko	Nagoya University
Nakamura, Hiroyuki	Kanazawa University
Nakamura, Koshi	Kanazawa Medical University
Nakamura, Mieko	Hamamatsu University School of Medicine
Nakamura, Motoyuki	Iwate Medical University
Nakamura, Yasuyuki	Kyoto Women’s University
Nakamura, Yosikazu	Jichi Medical University
Nakata, Yoshio	University of Tsukuba
Nakayama, Takeo	Kyoto University
Niino, Naoakira	J. F. Oberlin University
Nishimura, Kunihiro	National Cardiovascular and Cerebral Center
Nishino, Yoshikazu	Miyagi Cancer Center Research Institute
Nishiwaki, Yuji	Toho University
Noda, Hiroyuki	Harvard School of Public Health
Obara, Taku	Tohoku University
Ohira, Tetsuya	Osaka University
Ohshige, Kenji	Yokohama National University
Ojima, Toshiyuki	Hamamatsu University School of Medicine
Okada, Katsutoshi	Ehime University
Okamoto, Etsuji	National Institute of Public Health
Okamoto, Naoyuki	Kanagawa Cancer Center
Ooki, Syuichi	Ishikawa Prefectural Nursing University
Osaki, Yoneatsu	Tottori University
Oshima, Akira	Osaka Medical Center for Cancer and CVD
Ota, Atsuhiko	Fujita Health University School of Medicine
Ozasa, Kotaro	Radiation Effects Research Foundation
Pham, Truong-Minh	Radiation Effects Research Foundation
Pitiphat, Waranuch	Khon Kaen University
Rabito, Felicia	Tulane SPHTM
Sadakane, Atsuko	Jichi Medical University
Saito, Isao	Ehime University
Sakata, Kiyomi	Iwate Medical University
Sakurai, Masaru	Kanazawa Medical University
Sasazuki, Shizuka	National Cancer Center
Satoh, Toshihiko	Kitasato University
Seki, Ayumi	Tottori University
Sekikawa, Akira	University of Pittsburgh
Shibata, Akiko	Yamagata Prefectural Central Hospital
Shimazu, Akihito	The University of Tokyo
Shimazu, Taichi	National Cancer Center
Shimbo, Takuro	National Center for Global Health and Medicine
Shimouchi, Akira	The Research Institute of Tuberculosis
Shinkai, Shoji	Tokyo Metropolitan Institute of Gerontology
Shirai, Kokoro	Harvard Schoool of Public Health
Suzuki, Kohta	University of Yamanashi
Tabara, Yasuharu	Ehime University
Takatsuka, Naoyoshi	Gifu University
Takeshita, Tatsuya	Wakayama Medical University
Takeuchi, Ayano	The University of Tokyo
Takezaki, Toshiro	Kagoshima University
Tamakoshi, Akiko	Aichi Medical Uniersity
Tamashiro, Hiko	Hokkaido University
Tanaka, Hideo	Aichi Cancer Center
Tanaka, Junko	Hiroshima University
Tanaka, Keitaro	Saga University
Tanihara, Shinnichi	Fukuoka University
Teramukai, Satoshi	Kyoto University Hospital
Tsuda, Toshihide	Okayama University
Tsukuma, Hideaki	Osaka Medical Center for Cancer and CVD
Tsutsumi, Akizumi	University of Occupational and Environmental Health
Ueda, Kayo	National Institute for Environmental Studies
Wakai, Kenji	Nagoya University Graduate School of Medicine
Washio, Masakazu	St. Mary’s College
Watanabe, Makoto	National Cardiovascular Center
Watanabe, Shuichiro	J. F. Oberlin University
Wrishmeen, Sabawoon	The University of Tokyo
Yamada, Norio	Research Institute of Tuberculosis
Yamagata, Zentaro	University of Yamanashi
Yamagishi, Kazumasa	University of Tsukuba
Yamaji, Taiki	National Cancer Center
Yanagawa, Hiroshi	Health Promotion Research Center
Yanagita, Masahiko	Doshisha University
Yatsuya, Hiroshi	Nagoya University
Yoshiike, Nobuo	Aomori University of Health and Welfare
Yoshimura, Takesumi	Fukuoka Women’s University

